# Exploring the Characterization, Physicochemical Properties, and Antioxidant Activities of Chitosan-Encapsulated Green Tea Extract Microsphere Resin

**DOI:** 10.3390/polym17121633

**Published:** 2025-06-12

**Authors:** Lina Yu, Siyu Feng, Yu Song, Jie Bi, Yuan Gao, Luhui Wang, Chen Jiang, Mingqing Wang

**Affiliations:** 1 Shandong Peanut Research Institute, Qingdao 266100, China; lhtyln0626@163.com (L.Y.); songyuhss@163.com (Y.S.); bj.baby@163.com (J.B.); gaoyuan_hah@163.com (Y.G.); wlhfood@sina.com (L.W.); jiangchen_qd@126.com (C.J.); 2 College of Biotechnology, Jiangsu University of Science and Technology, Zhenjiang 212000, China; 13614505512@163.com

**Keywords:** chitosan-encapsulated green tea extract microsphere resin, preparation, characterization, physical properties, swelling properties, polyphenolic content, antioxidant activities

## Abstract

Chitosan, a naturally occurring alkaline polysaccharide with excellent biocompatibility, non-toxicity, and renewability, has the ability to undergo cross-linking reactions with polyphenolic compounds. In this study, chitosan-encapsulated green tea extract microsphere resin (CS-GTEMR) was successfully prepared using chitosan and green tea extract via reversed-phase suspension cross-linking polymerization. The structural characterization of CS-GTEMR was conducted using Fourier Transform Infrared Spectroscopy (FTIR) and Differential Scanning Calorimetry (DSC). Additionally, its physical properties, swelling behavior, polyphenol content, and antioxidant activities were investigated. The results indicate that CS-GTEMR consists of reddish-brown microspheres with a smooth surface and dense pores. The study found that the total content of polyphenolic compounds encapsulated in CS-GTEMR was 50.485 ± 0.840 μg/g. The characteristic absorption peak of phenolic hydroxyl groups appeared in the FTIR spectrum, suggesting that the polyphenolic compounds had been successfully encapsulated within the CS-GTEMR. The equilibrium swelling ratio of CS-GTEMR was determined to be 229.7%, indicating their suitability for use in solutions with a pH range of 1–13. In simulated gastric and intestinal fluids, the release rates of polyphenolic compounds from CS-GTEMR were 24.934% and 3.375%, respectively, indicating that CS-GTEMR can exert a sustained-release effect on polyphenolic compounds. CS-GTEMR demonstrated antioxidant activities such as scavenging DPPH radicals, superoxide anion radicals, hydroxyl radicals, and hydrogen peroxide, as well as exhibiting iron-reducing and molybdenum-reducing powers. With its high mechanical strength, acid resistance, and organic solvent resistance, CS-GTEMR can protect polyphenolic compounds from damage. Therefore, CS-GTEMR can be utilized as a natural antioxidant or preventive agent in food, expanding the application scope of green tea extracts.

## 1. Introduction

The important role of polyphenolic compounds in human health is of current interest. The main sources of polyphenolic compounds in diet are fruits and beverages, especially in tea and coffee [1,2,3]. Li et al. quantified 19 polyphenolic compounds from green tea extract by ultra-high performance liquid chromatography coupled with quadrupole-time of flight mass spectrometry (UPLC-Q-TOF MS) [4]. This suggests that tea products are a good source of polyphenolic compounds. Polyphenolic compounds generally have significant antioxidant and pharmacological capabilities, including scavenging free radicals, antibacterial, anti-inflammatory, and cholesterol-lowering activities. When polyphenolic compounds enter the plasma, they contribute to increasing the antioxidant capacity of the plasma and reduce the risk of cardiovascular disease by preventing the oxidation of plasma low-density lipoprotein. Rajapaksha et al. obtained eight polyphenolic compounds from *Schinus terebinthifolia* fruit, which exhibited significant antioxidant, cytotoxic, anti-inflammatory, analgesic, and antimicrobial properties, and also proliferation inhibition in MCF-7 human cancer cell lines [5]. However, the concentration and antioxidant activities of polyphenolic compounds are reduced in alkaline pH environments such as intestinal fluid in the human body, which greatly limits their application.

In order to solve the problem that polyphenolic compounds are easily lost in the human body, they can be encapsulated in polymers to reduce the influence of the pH value of the environmental solution on them. In this way, the release time of polyphenolic compounds can be controlled or prolonged, and their concentration levels in plasma can be maintained, so as to give full play to their physiological activities. Chitosan is a natural, non-toxic, non-immunogenic, alkaline polysaccharide that is biocompatible, renewable, and biodegradable. Due to the biocompatibility and adhesion properties of chitosan, bioactive substances can be encapsulated in chitosan, which contributes to the transport and sustained release of bioactive substances. Liu et al. incorporated inulin and chitosan into alginate-based microspheres for targeted delivery and release of quercetin into the colon [6]. Quercetin encapsulated in microspheres retained 80.3% after in vitro gastrointestinal digestion. Colonic fermentation experiments showed that quercetin release was delayed, but fermentation occurred within 3 h and was completely metabolized by microorganisms within 24 h. Yu et al. synthesized astaxanthin-loaded nanoparticles by the amidation reaction of carboxymethyl chitosan and methionine [7]. The loading capacity of astaxanthin reached 39.68 μg/mL, which realized the controllable release of astaxanthin in the simulated intestinal high-concentration ROS environment. In vitro cell experiments showed that the nanoparticles could effectively alleviate the oxidative damage caused by H_2_O_2_ on the intestinal epithelial cell line No.6 (IEC-6 cells). It is suggested that if polyphenolic compounds are encapsulated in chitosan microspheres, they will also have a controllable release effect and can give full play to the physiological activity of polyphenolic compounds.

Green tea extract, a product derived from the tea processing industry, primarily consists of polyphenolic compounds and also contains bioactive components such as tea polysaccharides and amino acids. It exhibits significant potential in promoting human health, with demonstrated effects including the inhibition of atherosclerosis, reduction in blood pressure, modulation of insulin secretion levels, enhancement of memory function, suppression of prostate cancer cell proliferation, and improvement of intestinal health. Hossain et al. conducted a multidimensional investigation encompassing cellular, animal, and molecular mechanistic studies to elucidate the effects of green tea extract on atherosclerosis [8]. Their findings demonstrated that green tea extract could mitigate oxidative stress and enhance cell survival rates while significantly improving the blood lipid profile in high-fat diet-fed mice. Molecular docking analysis revealed that catechin compounds exhibited strong binding affinities with key targets, including LOX-1, HMG-CoA reductase, caspase-3, and Nrf2. Meanwhile, de la Fuente-Muñoz et al. demonstrated that the combined extract of black tea and green tea effectively alleviated angiotensin II-induced cardiovascular dysfunction in mice through anti-inflammatory, antioxidant, and anti-apoptotic effects, while exhibiting antihypertensive properties [9]. These findings indicate that green tea extract may serve as a potential natural agent for preventing atherosclerosis and reducing cardiovascular risk. Sulaimani et al. revealed that green tea extract exerted no significant impact on postprandial blood glucose in healthy adults but delayed postprandial insulin secretion in the morning rather than at night [10]. This phenomenon untangles the time-dependent regulatory effect of green tea extract on insulin sensitivity. Joo et al. administered green tea extract to patients with subjective memory complaints for 12 weeks [11]. The results demonstrated enhanced memory function and strengthened functional connectivity within the default mode network, providing critical evidence for the cognitive-improving effects of green tea extract. In a separate study, Moalemi et al. treated PC3 prostate cancer cells with green tea extract for 48 h, revealing suppressed expression of cyclin B1, p-AR, CDK1, p-AKT, PSA, c-Myc, and p-CDK1 [12]. These findings offer a theoretical foundation for the design of antitumor treatment plans. In addition, green tea extract demonstrates remarkable efficacy in maintaining intestinal health. For instance, it significantly elevates the levels of antioxidant factors in the intestinal tract of mice while reducing the content of pro-inflammatory cytokines [13]. This intervention alleviates antibiotic-induced weight loss and intestinal pathological damage, promotes the colonization of *Bifidobacterium* and *Lactobacillus* genera in the murine gut microbiota, and concurrently inhibits the proliferation of *Escherichia coli* and *Enterococcus* species. The study investigating the effects of green tea extract on growth performance, *Clostridium perfringens* colonization, and inflammatory responses in broilers with experimental subclinical necrotic enteritis demonstrated that dietary supplementation with green tea extract alleviated characteristic growth suppression in chicks during subclinical necrotic enteritis, reduced the severity of *C. perfringens* infection in the intestine, and modulated inflammatory responses [14]. The aforementioned research findings comprehensively demonstrate the potent protective and health-enhancing capabilities of green tea extract in biological systems. It can be anticipated that green tea extract holds promising prospects as a potential natural therapeutic agent.

However, one critical issue warrants attention: among these diverse biological effects, green tea extract needs to exert its actions at distinct target sites, including but not limited to arterial tissues and the intestinal tract. If green tea extract can reach the target sites in the organism at sufficiently high concentrations, it can effectively exert its preventive and therapeutic effects. However, when administered in its current powdered or solution form, green tea extract is evidently unable to achieve the objective of reaching the target sites at high concentrations and functioning efficiently within the complex physiological environment of the organism. The development of an appropriate delivery system for green tea extract is crucial to achieve targeted release at specific sites with high bioavailability in vivo. Chitosan has emerged as the material of choice for in vivo green tea extract delivery due to its remarkable functional properties, excellent biocompatibility, and absence of toxic side effects. Bavi et al. fabricated chitosan–gelatin–green tea extract composite particles using an electrospray system [15]. The results demonstrated that these composite particles achieved sustained release of green tea extract for up to 9 days under pH 7.4 conditions, significantly enhanced cell viability, and effectively inhibited apoptosis. This system provides an excellent natural biomaterial for Parkinson’s disease treatment. Piran et al. encapsulated green tea extract in chitosan–citric acid nanoparticles, and their results showed that the antioxidant activity of tea polyphenols was enhanced [16]. This confirmed the potential of nanoparticles to deliver green tea polyphenols in food. Chuysinuan et al. investigated the controlled release behavior and antioxidant activity of green tea extract embedded in a composite of cyclodextrin and chitosan [17]. Through the evaluation of swelling properties, degradation curves, and the scavenging activities of DPPH and ABTS free radicals, it was proved that this method had a protective effect on green tea extract.

The above research results show that chitosan hydrogel has a protective effect on green tea extract and can exert its antioxidant activity. However, chitosan can only be dissolved under acidic pH conditions, which makes the preparation conditions of chitosan hydrogel extremely harsh. Thereby, the product of chitosan green tea extract is prone to degradation in physiological solutions. Kudłacik-Kramarczyk et al. evaluated the interaction between chitosan-encapsulated yellow tea extract hydrogel and simulated fluids by the change rule of pH value [18]. They found that incubation in simulated physiological solution led to a decrease in the contact angle of the hydrogel, which could even decrease by 60%. As can be seen, the direct encapsulation of green tea extract in chitosan hydrogel will affect its utilization rate in the organism. In order to overcome this disadvantage of chitosan hydrogel, chitosan should be modified to ensure that the prepared chitosan-encapsulated tea extract product has a stable structure and properties. Reversed-phase suspension cross-linking polymerization is an effective method for chitosan modification. When the cross-linking agent is added to the emulsion formed by chitosan acetate solution and liquid paraffin, the amino and hydroxyl groups in chitosan are cross-linked with the aldehyde group of the cross-linking agent, thus forming microspheres with dense surface and porosity. The cross-linking reaction can change the crystal structure of chitosan, making its products resistant to acids, alkalis, and chemicals. It is foreseeable that if the green tea extract is encapsulated in chitosan microspheres, the chitosan microspheres can resist the physiological fluid, allowing the green tea extract to exert its beneficial effects. In this paper, chitosan microspheres loaded with green tea extract were prepared by a reverse-phase suspension cross-linking polymerization method. The morphology was observed by a high-resolution three-dimensional microscope, and the size distribution was analyzed by a particle size analyzer. The structural characteristics of the polyphenolic compounds in the microspheres combined with the chitosan matrix were analyzed by FTIR and DSC. The feasibility of the reverse-phase suspension cross-linking polymerization method in the application of chitosan microspheres encapsulation of green tea extract was explored by evaluating the swelling properties of microspheres, the release properties, and the antioxidant activities of polyphenolic compounds. Compared with existing studies on chitosan-encapsulated polyphenolic compounds microspheres, the novelty of this research involves designing a reverse-phase suspension two-step cross-linking polymerization method for preparing chitosan-encapsulated green tea extract microspheres. The advantage of this approach is that a hydrogel is first formed through pre-cross-linking, followed by a secondary cross-linking reaction to generate stable and robust chitosan-encapsulated green tea extract microspheres. They exhibit the microstructural characteristics of the microspheres, demonstrating resistance to acids, alkalis, and chemicals, thereby facilitating more controlled release of the encapsulated compounds. Given the superior antioxidant activity of green tea extract and its significant potential in protecting and enhancing organismal health, this study aimed to develop a chitosan-encapsulated green tea extract microsphere resin (CS-GTEMR) capable of protecting and controllably releasing polyphenolic compounds. The primary objective of this research was to establish CS-GTEMR as a promising natural therapeutic agent for the prevention and treatment of certain diseases, as well as a nutraceutical supplement to safeguard human health. Furthermore, CS-GTEMR could be developed into a functional food ingredient for meat products, baked goods, and beverages, where it would serve as a natural antioxidant to extend shelf life and thereby broaden the application scope of green tea extract.

## 2. Materials and Methods

### 2.1. Chemical Reagents

Chitosan with a viscosity average molecular weight of 5.3 × 10^5^ and deacetylation degree (DDA) 85% was provided by Lizhong Chitosan Co., Ltd., Qingdao, China, and used without any purification. Green tea extract (GTE) was obtained from Hainan Qunli Pharmaceutical Co., Ltd., Sanya, China. Acetic acid, liquid paraffin, ethyl acetate, Span-80, formaldehyde solution, 50% glutaraldehyde solution, petroleum ether (boiling range 60–90 °C), acetone, and anhydrous ethanol were analytical reagents produced by Sinopharm Chemical Reagent Co., Ltd., Shanghai, China. All other reagents were of analytical grade and used as received.

### 2.2. Preparation of CS-GTEMR and Chitosan Microsphere Resin (CS-MR)

The preparation of CS-GTEMR by using reversed-phase suspension cross-linking polymerization technology was carried out according to the method of Yu et al. [19]. The process involves 8 steps, which are dissolution, dispersion, emulsification, pre-cross-linking, cross-linking, filtration, washing, and drying. First, green tea extract (0.5% *w*/*v*) was dissolved in distilled water, and then acetic acid (2% *v*/*v*) was added to the green tea extract solution with stirring. Chitosan (5% *w*/*v*) was added to the mixture with a glass rod and stirred to form a viscous red-brown chitosan green extract mixture with many bubbles. After standing overnight, the mixed liquid is degassed in a vacuum. Secondly, the above-mentioned mixed liquid was added to liquid paraffin (50% *v*/*v*). The mixed liquid was stirred in a water bath at 40 °C at 300 r/min for 20 min to form a suspension with many small liquid droplets. Thirdly, Span-80 (0.6% *w*/*v*) was added to the suspension, followed by the addition of ethyl acetate (10% *v*/*v*). In a water bath at 40 °C, the mixed liquid was stirred at 300 r/min for 20 min to form an emulsion. Fourthly, formaldehyde solution (10% *v*/*v*) was added to the emulsion, in a water bath at 50 °C, and the emulsion was stirred at 300 r/min for 20 min. Fifth, after adding the glutaraldehyde solution (5% *v*/*v*) to the above solution, the stirring speed was immediately reduced to 175 r/min, and the temperature was increased to 60 °C. The pH value of the reaction mixture was adjusted to 7.5 with 2 mol/L NaOH solution, and then stirred for 3 h. Sixth, when the reaction is over, a proper amount of petroleum ether is poured into the reaction mixture and mixed evenly. After CS-GTEMR settling, discard the upper layer of liquid. Then, pour in an appropriate amount of petroleum ether, mix evenly, and remove the petroleum ether with a vacuum filter to obtain CS-GTEMR. Seventh, wash the CS-GTEMR with acetone, anhydrous ethanol, and distilled water in turn. Then, CS-GTEMR was soaked in distilled water, and after CS-GTEMR settled to the bottom of the container, the upper layer of distilled water and CS-GTEMR suspended on the surface was decanted. The process was repeated five times. Finally, the CS-GTEMR was dried under vacuum at 50 °C.

CS-MR was also prepared by reversed-phase suspension cross-linking polymerization technology. When preparing CS-MR, chitosan (5% *w*/*v*) was added directly to the acetic acid solution (2% *v*/*v*) instead of the green tea extract acetic acid solution. The other steps were the same as the preparation method of CS-GTEMR.

### 2.3. Determination of Physical Properties

Physical properties of CS-GTEMR and CS-MR were determined according to the method of Yu et al. [20].

#### 2.3.1. Determination of Water Absorption Capacity

The 0.1 g samples were fully swelled in distilled water and filtered. Then, the surface water of the samples was sucked dry and weighed (*W*_1_, g). Finally, the samples were dried at 105 °C for 4 h and weighed (*W*_2_, g). The water absorption capacity (*M*, %) was calculated using Formula (1).

(1) $$M(\%)=\frac{W_{1}-W_{2}}{W_{1}}\times 100$$

#### 2.3.2. Determination of Pile-Up Density

Pile-up density is the weight of the samples per unit volume, in which the volume includes the skeleton, pore, and interstitial volume of the samples. Take a sample of volume 2 mL (*V_P_*, mL) and weigh its mass (*W*, g). The pile-up density (*ρ_p_*, g/mL) was calculated using Formula (2).

(2) $$\rho_{P}(g/mL)=\frac{W}{V_{P}}$$

#### 2.3.3. Determination of Skeleton Density

Add 5 mL of n-heptane to a 10 mL graduated cylinder and measure its mass (*W*_1_, g). Then, pour out the n-heptane. Add 0.1 g (*W*, g) of the sample into the cylinder, followed by 2 mL of n-heptane, and let it sit for 2 h. Afterward, top up the cylinder with n-heptane to the 5 mL mark and measure its mass (*W*_2_, g) again. The skeletal volume (*V_T_*, cm^3^) and skeletal density (*ρ_T_*, g/cm^3^) can be calculated using Formulas (3) and (4), respectively.

(3) $$V_{T}=\frac{W_{1}-W_{2}+W}{dt}$$

where *dt* is the n-heptane density, 0.6830 g/cm^3^.

(4) $$\rho_{T}(g/cm^{3})=\frac{W}{V_{T}}$$

#### 2.3.4. Determination of Pore Degree

The pore degree (*P*) can be calculated using Formula (5), where *ρ_T_* and *M* are skeletal density and water absorption capacity, respectively.

(5) $$P=\frac{\rho_{T}M}{\rho_{T}M+1-M}$$

#### 2.3.5. Determination of Free Aldehyde Group Content

After fully swelling 0.1 g (W, g) of the sample in water, the water was removed by suction filtration. Then, 10.0 mL of hydroxylamine reagent was added, and the mixture was oscillated in a 25 °C water bath for 1 h. Following this, two drops of 0.05% bromophenol blue indicator were added, and the solution was titrated to the endpoint with a standard hydrochloric acid solution (N, 0.02 mol/L). The volume of standard hydrochloric acid consumed by the blank control was denoted as *V*_0_ (mL), while the volume consumed by the sample was denoted as *V*_1_ (mL). The free aldehyde group content was calculated using Formula (6).

(6) $$\mathrm{Free}\;\mathrm{aldehyde}\;\mathrm{group}\;\mathrm{content}\;(\mathrm{mmol}/g)=\frac{N\times (V_{0}-V_{1})}{W}$$

#### 2.3.6. Determination of Weak Basic Exchange Capacity

After fully swelling 0.1 g (*W*, g) of the sample in water, it was filtered to remove moisture, and then oscillated with 20.0 mL of standard hydrochloric acid solution (*N*_1_, 0.05 mol/L) in a 25 °C water bath for 1 h. Following this, 15.0 mL of the supernatant was taken, and two drops of 0.2% phenolphthalein indicator were added. The solution was then titrated to the endpoint using a standard sodium hydroxide solution (*N*_2_, 0.05 mol/L). The volume of standard hydrochloric acid solution consumed was denoted as *V*_1_ (mL), and the volume of standard sodium hydroxide solution consumed was denoted as *V*_2_ (mL). The weak basic exchange capacity was calculated using Formula (7) below.

(7) $$\mathrm{Weak}\;\mathrm{basic}\;\mathrm{exchange}\;\mathrm{capacity}\;(\mathrm{mmol}/g)=\frac{N_{1}\times V_{1}-N_{2}\times V_{2}\times \frac{20}{15}}{W}$$

### 2.4. Structure and Thermal Stability Characterization

The morphological characterization of the sample was carried out by a super depth of field three-dimensional microscopy system (VHX-950F, KEYENCE, Osaka, Japan). Average particle diameter and particle size distribution of the samples were determined by a laser diffraction particle size analyzer (LS200, Beckman Coulter, Inc., Fullerton, CA, USA). The FTIR spectra of samples were determined by a Fourier transform infrared spectrometer (Nicolet NEXUS 470, Thermo Nicolet Corporation, Madison, WI, USA). DSC curves of samples were determined by differential scanning calorimeter (200PC, NETZSCH-Gerätebau GmbH, Selb, Germany), and their thermal stability was analyzed.

### 2.5. Determination of the Polyphenolic Compounds Content

Polyphenolic compounds content in CS-GTEMR was determined according to the method of Pérez et al. [21]. The 0.1 g sample was fully swollen in distilled water and then filtered to remove excess water. A total of 0.5 mL of Folin–Ciocalteu reagent was added to the sample, mixed well, and allowed to stand in the dark for 5 min. Subsequently, 2.5 mL of a Na_2_CO_3_ solution (20% *w*/*v*) was added to the mixture, which was then mixed uniformly and left to stand in the dark for 1 h. The absorbance was determined at 725 nm, and the polyphenolic compounds content of the sample was calculated based on a standard curve (pyrogallol). The reported polyphenolic compounds content represents the Mean ± SD of three independent experiments.

### 2.6. Determination of Antioxidant Activities

#### 2.6.1. Determination of DPPH Free Radical Scavenging Activity

DPPH free radical scavenging activity was determined according to the method of Yu et al. [22]. Three groups of different reaction solutions were prepared, involving solution 1 (2 mL sample solution and 2 mL DPPH solution (0.2 mmol/L)), solution 2 (2 mL sample solution and 2 mL anhydrous ethanol), and solution 3 (2 mL DPPH solution (0.2 mmol/L) and 2 mL distilled water). All three mixture solutions were kept in the dark at room temperature for 20 min. Then, the absorbance values of *A_i_* (solution 1), *A_j_* (solution 2), and *A*_0_ (solution 3) were determined at 517 nm. The DPPH free radical scavenging rate (8) was calculated as follows:

(8) $$\mathrm{DPPH}\;\mathrm{free}\;\mathrm{radical}\;\mathrm{scavenging}\;\mathrm{rate}\;(\%)=(1-\frac{A_{i}-A_{j}}{A_{0}})\times 100$$

#### 2.6.2. Determination of Superoxide Anion Free Radical Scavenging Activity

Superoxide anion free radical scavenging activity was determined according to the method of Zhang et al. [23]. To a 2.0 mL Tris-HCl buffer solution (pH 8.2, 0.1 mmol/L), add 2.0 mL of distilled water, mix well, and let it stand in a water bath at 25 °C for 20 min. Then, add 0.1 mL of preheated 3 mmol/L pyrogallol at 25 °C, mix well, and immediately measure the absorbance at 325 nm every 30 s for a total of 11 measurements. Calculate the rate of change, Δ*A*_0,_ of pyrogallol absorbance over time. A total of 2.0 mL of sample solution was mixed with 2.0 mL of distilled water and allowed to stand at 25 °C for 20 min after thorough mixing. Then, 0.1 mL of 3 mmol/L pyrogallol, preheated in a 25 °C water bath, was added. The mixture was immediately measured for absorbance at 325 nm every 30 s, with a total of 11 measurements taken. The rate of change in absorbance (Δ*A*_S_) of the sample solution over time was calculated. The superoxide anion radical scavenging rate *E* (%) was determined using Formula (9).

(9) $$E(\%)=\frac{\Delta A_{0}-\Delta A_{S}}{\Delta A_{0}}\times 100$$

#### 2.6.3. Determination of Hydroxyl Radical Scavenging Activity

Hydroxyl free radical scavenging activity was determined according to the method of Falcone et al. [24]. (1) Mix 2 mL of phosphate buffer (pH = 7.4, 0.1 mol/L), 1 mL of distilled water, and 1 mL of FeSO_4_ solution (0.75 mmol/L) uniformly. Then, add 1 mL of H_2_O_2_ (0.01%) and mix for 1 min before adding 1 mL of 1,10-phenanthroline anhydrous ethanol solution (0.75 mmol/L). Allow the mixture to stand in a water bath at 37 °C for 60 min. Determine the absorbance value *A_P_* at 536 nm;

(2) Replace the H_2_O_2_ in step (1) with 1 mL of distilled water, keeping all other conditions the same as in (1), and measure the absorbance value *A_B_*;

(3) Substitute the distilled water in step (1) with 1 mL of sample solution, and measure the absorbance value *A_S_*. Calculate the hydroxyl radical scavenging rate *D* (%) according to Formula (10).

(10) $$D(\%)=\frac{A_{S}-A_{P}}{A_{B}-A_{P}}\times 100$$

#### 2.6.4. H_2_O_2_ Scavenging Activity

H_2_O_2_ scavenging activity was determined according to the method of Porcher et al. [25]. After fully swelling CS-GTEMR (0.1 g) in distilled water, remove the water and add 4 mL of H_2_O_2_ solution (2 mmol/L). Let the mixture stand for 10 min. Adjust the zero point with phosphate buffered solution (pH = 7.4) and determine the absorbance values of the H_2_O_2_ solution and the sample at 203 nm.

#### 2.6.5. Determination of Iron Reducing Power

Iron reducing power was determined according to the method of Yu et al. [22]. A total of 2.5 mL phosphate buffer solution (pH 6.6, 0.1 mol/L) and 2.5 mL K_3_Fe(CN)_6_ (1%) were added to 1.0 mL sample solution, mixed evenly, and stood in a water bath at 50 °C for 20 min. Next, 2.5 mL TCA (10%) was added to the above mixture solution and centrifuged for 5 min at 2800× *g*. Then, 2.5 mL distilled water and 0.5 mL FeCl_3_ (0.1%) were added to 2.5 mL supernatant, mixed evenly, and the mixture solution stood at room temperature for 10 min. The effect of iron reducing power was determined by the absorbance value at 700 nm.

#### 2.6.6. Determination of Molybdenum Reducing Power Activity

Molybdenum reducing power was determined according to the method of Yu et al. [22]. After fully swelling CS-GTEMR (0.1 g) in distilled water and removing the excess water, 4 mL of phosphomolybdenum blue reagent solution was added. As a control, 0.1 mL of ascorbic acid solution (100 μg/mL) was added to 4 mL of phosphomolybdenum blue reagent solution. Both the sample and the control were incubated in a water bath at 95 °C for 90 min, then cooled to room temperature, and their absorbance values were determined at 695 nm. *A_S_* is the sample’s absorbance, and *A_C_* is the control’s absorbance. The molybdenum reducing power activity was calculated according to Formula (11).

(11) $$\mathrm{Molybdenum}\;\mathrm{reducing}\;\mathrm{power}\;\mathrm{activity}\;(\%)=\frac{A_{C}-A_{S}}{A_{C}}\times 100$$

### 2.7. Statistical Analysis

All experiments were performed in triplicate, and data were expressed as means and standard deviations. The SPSS Statistics 17.0 software (IBM Inc., Armonk, NY, USA) was used to analyze the variance of the results with the method of least significant difference (LSD).

## 3. Results and Discussion

### 3.1. Characterization of CS-GTEMR

#### 3.1.1. Basic Physical Properties of CS-GTEMR

**Figure 1:**
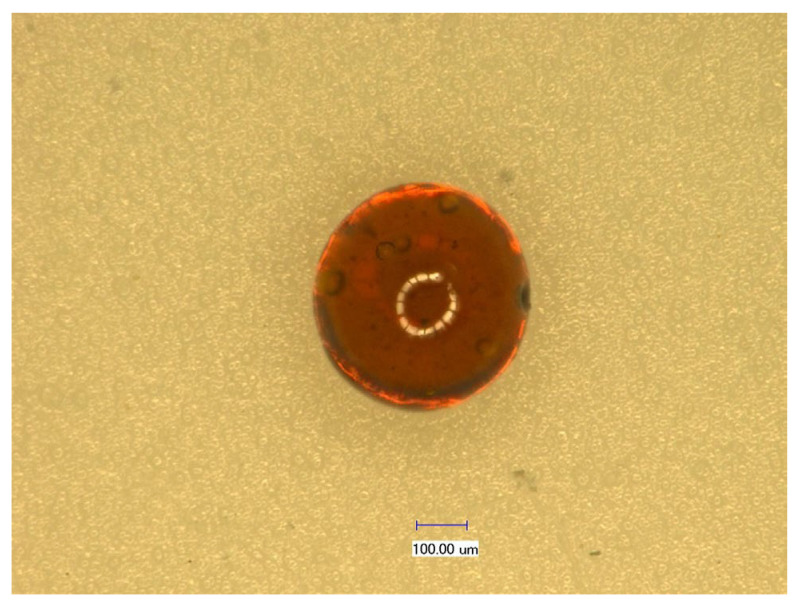
Microphotograph of CS-GTEMR (×100).

**Figure 2:**
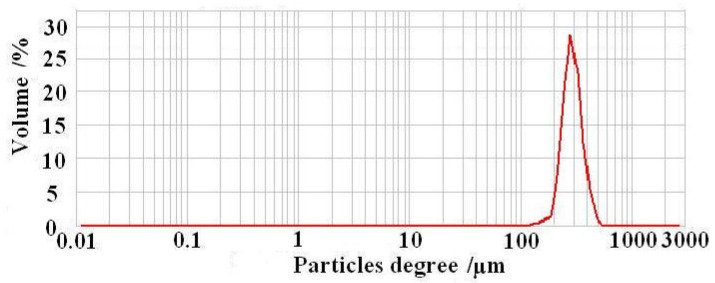
Particle size distribution of CS-GTEMR.

The three-dimensional super depth of field microscopy image of CS-GTEMR is presented in Figure 1. CS-GTEMR appears as a reddish-brown, spherical body with a smooth surface and a dense, porous structure. This configuration enhances their specific surface area, favoring the exertion of active effects. During the preparation of CS-GTEMR via the reversed-phase suspension cross-linking polymerization technology, chitosan and green tea extract acetic acid solution are introduced into liquid paraffin containing emulsifier and pore-forming agent, resulting in the formation of an oil-in-water emulsion. In the traditional preparation method of chitosan microsphere gel, only one cross-linking reaction is performed using cross-linking agents such as glutaraldehyde, epichlorohydrin, or genipin [26,27]. However, in this study, formaldehyde was used as a pre-cross-linking agent to form a liquid gel of chitosan. Subsequently, glutaraldehyde was employed as the cross-linking agent, and after a secondary cross-linking reaction, the chitosan liquid gel solidified into a hard and porous microsphere resin. Furthermore, during the secondary cross-linking process, the polyphenolic compounds and polysaccharides present in green tea extract can effectively interact with chitosan, resulting in the formation of chitosan-encapsulated green tea extract microsphere resin (CS-GTEMR). The determination of particle size distribution revealed that the average particle size of CS-GTEMR is approximately 316.106 µm, with 100% of the particles being smaller than 549.541 µm. Additionally, 90%, 50%, and 10% of the particles are smaller than 460.905 µm, 309.487 µm, and 237.500 µm, respectively. Based on the particle size distribution diagram in Figure 2, it can be observed that the particle size of CS-GTEMR follows a normal distribution with a relatively uniform distribution. There exist interactions between the chitosan matrix and components such as tea polyphenols from green tea extracts, resulting in smaller particle sizes. These hydrophilic interactions are essential forces for the formation of spherical particles. Additionally, some covalent bonds can also be formed between them; for instance, the amino groups in chitosan can react with the quinone rings in tea polyphenols. These physicochemical interactions have been confirmed in proteins and polyphenols [28].

Table 1 presents the physical properties of CS-GTEMR. Yu et al. reported the physical properties of chitosan microsphere resin (CS-MR) prepared by reversed-phase suspension cross-linking polymerization technology [20]. The water adsorption capacity of CS-MR is 51.982 ± 1.944%, indicating that CS-GTEMR exhibits a higher water adsorption capacity. This enhanced capacity is attributed to the presence of green tea extract components in CS-GTEMR. Among these components, groups such as hydroxyl groups from polyphenolic compounds or polysaccharides, amino groups, and carboxyl groups from proteins or amino acids readily bond with water, thereby increasing the water adsorption capacity [29]. The content of free aldehyde groups in CS-GTEMR is lower than that in CS-MR (0.315 ± 0.009 mmol/g). During the cross-linking reaction, the proteins and amino acids, which are the main components of green tea extract, contain free amino groups. These amino groups have the potential to react with either both aldehyde groups of glutaraldehyde or with just one aldehyde group (where the other aldehyde group has already cross-linked with the amino group of chitosan), resulting in the formation of carbon–nitrogen double bonds (Schiff base) [30]. This process contributes to a reduction in free aldehyde groups in CS-GTEMR. The weak basic exchange capacity of CS-GTEMR is greater than that of CS-MR (1.311 ± 0.084 mmol/g). In addition to the free amino groups present in chitosan, the proteins and amino acids from green tea extract in CS-GTEMR also contain amino and carboxyl groups, all of which contribute to its weak basic exchange capacity. Hence, CS-GTEMR exhibits a higher weak basic exchange capacity. Based on the above analysis, CS-GTEMR can be described as a reddish-brown, spherical, porous microsphere resin with water absorption and weak basic exchange capabilities.

#### 3.1.2. FTIR Spectroscopy Analysis

**Figure 3:**
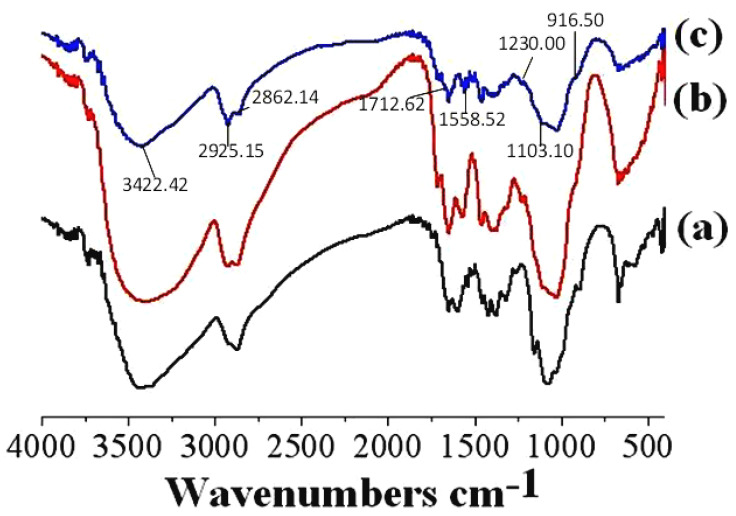
FTIR spectrum (**a**) chitosan powder, (**b**) CS-MR, and (**c**) CS-GTEMR.

Figure 3 presents the FTIR spectra of chitosan powder, CS-MR, and CS-GTEMR, with the wave numbers of their main peaks listed in Table 2. The broad peak, formed by the overlapping of the stretching vibration absorption peaks of ν(O-H) and ν(N-H) in chitosan powder, CS-MR, and CS-GTEMR, shifts from 3444 cm^−1^ to approximately 3420 cm^−1^ towards the shorter wave number. This shift is attributed to the presence of the Schiff base П bond in CS-MR and CS-GTEMR, which induces a weak association between the -OH hydrogen and the П electron cloud, resulting in a red shift of the hydroxyl stretching vibration peak [31]. Both CS-MR and CS-GTEMR exhibit characteristic absorption peaks of aldehyde groups around 1716 cm^−1^, indicating the presence of free aldehyde groups from glutaraldehyde that did not react with amino groups [32]. Although the proteins and amino acids, which are the main components of green tea extract in CS-GTEMR, contain free amino groups that can react with glutaraldehyde to form Schiff bases, their low concentrations are insufficient to completely eliminate the pendant aldehyde groups, only reducing their amount. The disappearance of the amino bending vibration absorption peak at 1650 cm^−1^ and the emergence of a Schiff base absorption peak at 1558 cm^−1^ in CS-GTEMR indicate the formation of a -C=N bond through the cross-linking reaction between glutaraldehyde and amino groups. This finding aligns with previous reports stating that Schiff bases primarily form between 1540 and 1590 cm^−1^ [33]. Since the proteins and amino acids in CS-GTEMR contain free amino groups that can react with glutaraldehyde to form Schiff bases, the amide II peak in the FTIR spectrum of CS-GTEMR remains unchanged. After the chitosan cross-linking reaction, the intensity of the ν(C-O) stretching vibration absorption peak of their C_6_-OH groups is significantly reduced. The possible reason is that the chitosan molecules undergo a structural transformation from a long-chain structure to a coiled and curly spatial structure after the cross-linking reaction. The primary hydroxyl groups may form hydrogen bonds with nitrogen or hydrogen atoms. Additionally, the high reactivity of the primary hydroxyl groups allows them to bond with the aldehyde group of glutaraldehyde. The characteristic absorption peak of β-D-glucopyranoside in CS-GTEMR has shifted to 916 cm^−1^, indicating that the cross-linking reaction did not result in the ring-opening of the β-D-glucopyranose ring. Instead, it is likely that the amino groups in CS-GTEMR formed hydrogen bonds with the hydroxyl groups of chitosan, strengthening the force between glucose units in chitosan and leading to a change in the peak position. The presence of a characteristic absorption peak of phenolic hydroxyl groups, ν(H-O), at 1230 cm^−1^ in CS-GTEMR suggests the existence of polyphenolic compounds within the material [34].

#### 3.1.3. Differential Scanning Calorimetry (DSC) Analysis

**Figure 4:**
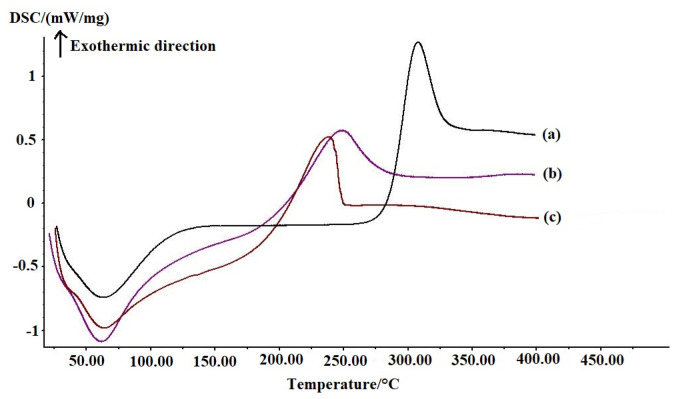
DSC of (**a**) CS-GTEMR, (**b**) CS-MR, and (**c**) chitosan powder.

Figure 4 displays the DSC curves of chitosan powder, CS-MR, and CS-GTEMR. The first heating stage curve reveals that the onset temperature of the endothermic peak for CS-GTEMR is 42.95 °C, with a peak end temperature of 91.34 °C and an endothermic enthalpy ΔH of 42.02 J/g. In comparison, the experimental data for chitosan powder and CS-MR are 57.06 °C, 82.88 °C, and 40.49 J/g, and 39.93 °C, 87.74 °C, and 63.41 J/g, respectively. The endothermic peak observed in this stage is primarily attributed to the dehydration of chitosan macromolecules [35]. The maximum endothermic peak of CS-GTEMR is greater than that of chitosan powder and CS-MR, while its endothermic enthalpy lies between that of chitosan powder and CS-MR. Most of the water molecules in chitosan powder are bonded to amino groups, forming hydrogen bonds, which require less energy to break. When chitosan is prepared into spherical cross-linked resin, some of its amino groups react with glutaraldehyde to form Schiff bases. The water molecules originally bonded with amino groups are then bonded with hydroxyl groups. This requires more energy to break the hydrogen bonds formed between water molecules and hydroxyl groups, thus explaining why the endothermic enthalpy of CS-MR is higher than that of chitosan powder. In CS-GTEMR, besides bonding with hydroxyl groups, water molecules also bond with the free amino groups of proteins and amino acids. The energy required to break the hydrogen bonds formed between amino groups and water molecules is less than that required to break the hydrogen bonds between water molecules and hydroxyl groups. Therefore, the endothermic enthalpy of CS-GTEMR is greater than that of chitosan powder but less than that of CS-MR.

The second heating stage revealed that the exothermic peak of CS-GTEMR initiated at 202.69 °C and terminated at 247.40 °C, with an exothermic enthalpy of 85.96 J/g. The experimental data for chitosan powder and CS-MR were as follows: 288.58 °C, 326.09 °C, 124.20 J/g, and 215.36 °C, 276.68 °C, 85.63 J/g, respectively. The exothermic peak in this stage is associated with the thermal and oxidative decomposition of chitosan, the thermal decomposition of polysaccharides, polyphenolic compounds, proteins, and amino acids, as well as the volatilization and elimination of volatile products. The thermal degradation temperatures decrease in the order of chitosan powder, CS-MR, and CS-GTEMR. Chitosan exhibits two types of intramolecular hydrogen bonds: one between the hydroxyl group at carbon 3 and the oxygen atom at carbon 5, and the other between the hydroxyl group at carbon 6 and the nitrogen atom of the amino group. Due to its good crystallinity, chitosan demonstrates excellent thermal stability [36]. However, when chitosan is prepared into cross-linked spherical resins, the formation of Schiff bases between amino groups and glutaraldehyde disrupts the intramolecular hydrogen bonds within the chitosan chains. The decrease in the crystallinity of chitosan affects its thermal stability, leading to the conclusion that the thermal stability of CS-MR is lower than that of chitosan powder. When green tea extract is added during the cross-linking process, the hydroxyl and amino groups present in the extract further disrupt the hydrogen bonds within the chitosan chains, resulting in the thermal stability of CS-GTEMR being even lower than that of CS-MR. Additionally, DSC curve data reveal the glass transition temperature ranges for chitosan powder, CS-MR, and CS-GTEMR to be 290.05~300.60 °C, 219.59~223.22 °C, and 192.78~211.18 °C, respectively. These results confirm that the thermal stability of the three substances, from highest to lowest, is chitosan powder, CS-MR, and CS-GTEMR.

### 3.2. Results and Analysis of Swelling Properties of CS-GTEMR

#### 3.2.1. Results and Analysis of CS-GTEMR Swelling Properties in Water

**Figure 5:**
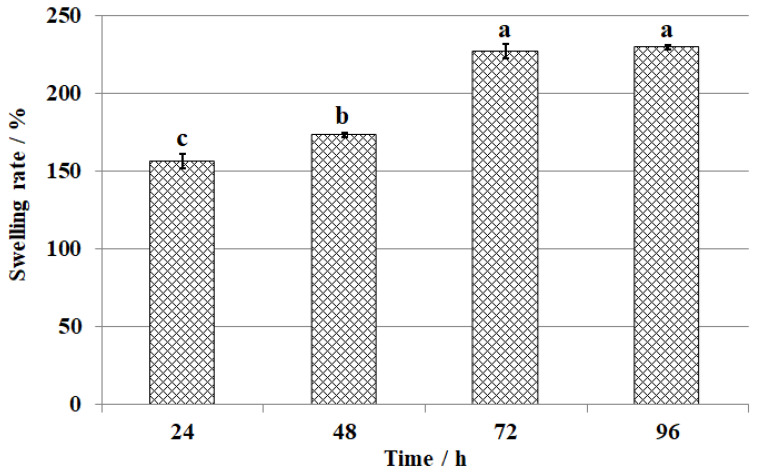
Swelling kinetic curves of CS-GTEMR in water (different letters in each indicator mean significant differences at 0.05 level (*p* < 0.05, *n* = 3)).

The swelling ratio of CS-GTEMR in distilled water at different times is shown in Figure 5. CS-GTEMR exhibits swelling properties in distilled water, with an equilibrium swelling ratio of 229.7%. Zhu et al. investigated the swelling behavior of tannic acid-modified keratin/sodium alginate/carboxymethyl chitosan biocomposite hydrogels [37]. The results indicated that the modified hydrogels exhibited a three-dimensional microporous structure with a swelling ratio of 1541.6%. However, the CS-GTEMR prepared in this study appeared spherical in shape and had a more compact texture, resulting in a lower swelling ratio compared to the aforementioned findings. The main components of green tea extract contained in CS-GTEMR mostly possess hydroxyl and amino groups, which easily bond with water molecules, thereby increasing the water absorption and swelling ratio. This result aligns with the high water absorption capacity and porous physical properties of CS-GTEMR. As illustrated in Figure 5, no statistically significant difference (*p* > 0.05) was observed in the swelling ratio of CS-GTEMR between 72 h and 96 h, indicating that the swelling equilibrium had been achieved by 72 h. Extending the swelling time further will not increase the swelling ratio. This phenomenon indicates that the microsphere resin prepared by reversed-phase suspension cross-linking polymerization technology has a tough structure and will not rupture due to excessive swelling in water. This suggests that CS-GTEMR can be applied in aqueous environments.

#### 3.2.2. Results and Analysis of CS-GTEMR Swelling Properties in Different pH Solutions

**Figure 6:**
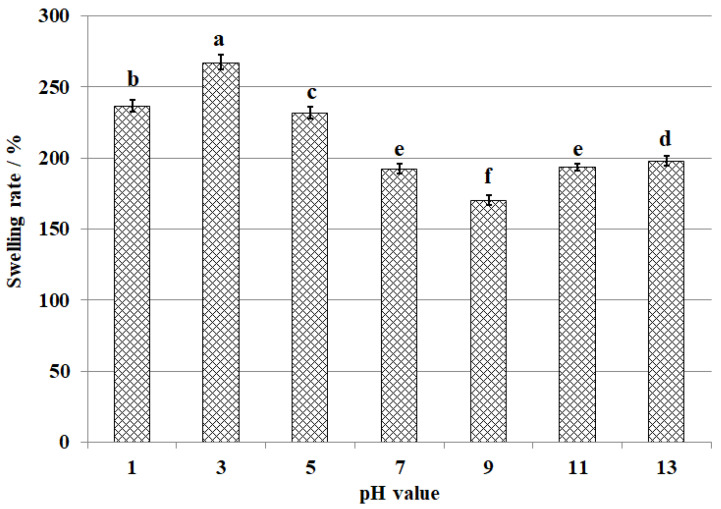
Swelling rates of CS-GTEMR in different pH solutions (different letters in each indicator mean significant differences at 0.05 level (*p* < 0.05, *n* = 3)).

The equilibrium swelling ratios of CS-GTEMR in buffer solutions with different pH values after 96 h of swelling are presented in Figure 6. Upon completion of the swelling process, no discoloration or microsphere damage was observed in the CS-GTEMR samples across all pH buffers, indicating that CS-GTEMR can maintain a stable structural state in solutions with pH values ranging from 1 to 13, and is capable of absorbing water and swelling under these conditions. Notably, the equilibrium swelling ratio of CS-GTEMR reached a minimum value of 170.2% in a Tris-HCl buffer at pH 9.0 (*p* < 0.05). The maximum equilibrium swelling ratio of the material in a disodium hydrogen phosphate–citric acid buffer solution at pH 3.0 is 267.5% (*p* < 0.05). It should be noted that no significant difference was observed in swelling ratios between the pH 7 and pH 11 groups (*p* > 0.05). However, statistically significant differences were identified between the swelling ratios at pH 13 versus those at both pH 7 and pH 11 (*p* < 0.05). The active hydroxyl and amino groups of polyphenolic compounds, polysaccharides, proteins, and amino acids from green tea extract in CS-GTEMR can bond with water molecules under different pH conditions, resulting in varying equilibrium swelling ratios. These findings suggest that CS-GTEMR can also be applied in solutions with a pH range of 1–13.

### 3.3. Evaluation of the Polyphenolic Compounds in CS-GTEMR

#### 3.3.1. Content of Polyphenols in CS-GTEMR

The content of polyphenolic compounds encapsulated in CS-GTEMR was determined using the Folin–Ciocalteu method, and the measured value was 50.485 ± 0.840 μg/g. Chitosan is a linear polymer composed of multiple linked glucosamine units. Under acidic pH conditions, it exhibits a high positive charge density due to the presence of a positive charge on each glucosamine unit. Polyphenolic compounds with specific molecular weights and sizes can serve as complexing agents. The coordination interaction between polyphenolic compounds and chitosan can be either reversible or irreversible. The reversible coordination of polyphenolic compounds and chitosan occurs in two stages. In the first stage, polyphenolic compounds and chitosan form soluble complexes through non-covalent bonding, achieving an equilibrium state in solution. In the second stage, the equilibrium state is disrupted, leading to the aggregation and precipitation of these soluble complexes from the solution. This entire process is typically reversible, and under appropriate conditions, the precipitated complexes can be redissolved. In this study, prior to the preparation of CS-GTEMR, a mixture of chitosan and green tea extract was dissolved in an acetic acid solution. During the dissolution process, feather-like aggregates were observed in the mixed solution of chitosan and green tea extract, indicating the formation of reversible complexes between chitosan and the polyphenolic compounds present in the green tea extract. In the emulsification step of the microsphere preparation process, the complexes precipitated from the polyphenolic compounds and chitosan were redissolved to form soluble complexes. After undergoing a secondary cross-linking reaction, polyphenolic compounds are encapsulated within chitosan microspheres in a stable coordination structure. Evidently, this reversible complex dissolution process facilitates the encapsulation of a greater number of polyphenolic compounds within the chitosan microspheres. Furthermore, the enhanced stability of the polyphenolic compounds’ binding state within the microspheres is beneficial for their controlled release and the exertion of their active effects during application. During the preparation of chitosan-encapsulated olive leaf extract microspheres using the spray drying method, a soluble complex is formed between the olive leaf extract and the chitosan matrix. If the interaction between the extract and chitosan mixture does not progress to the second stage of aggregation during the spray drying process, the resulting microspheres will contain a low amount of olive leaf extract.

#### 3.3.2. Results and Analysis of the Polyphenolic Compounds Release Rate in Simulated Gastric and Intestinal Fluid

When exposed to simulated gastric fluid and simulated intestinal fluid, the release rates of polyphenolic compounds from CS-GTEMR were 24.934 ± 0.168% and 3.375 ± 0.134%, respectively. It can be inferred that the release rate of polyphenolic compounds is higher in simulated gastric fluid compared to simulated intestinal fluid. The swelling ratios of CS-GTEMR at various pH values obtained in this study indicate that within the pH range of 1–3, which corresponds to simulated gastric fluid, the swelling ratio of CS-GTEMR is significantly higher than at other pH values. In this highly acidic environment, the porous structure of CS-GTEMR exhibits a high swelling ratio, resulting in the exposure of a significant amount of encapsulated polyphenolic compounds to the simulated gastric fluid. Under these conditions, a large number of hydrogen ions may competitively displace the amino groups of chitosan that were originally covalently coordinated with the polyphenolic compounds. Based on the reversible coordination mechanism between polyphenolic compounds and chitosan (as described in Section 3.3.1), under acidic conditions, some of the aggregated and precipitated complexes redissolve and subsequently dissociate into the ligand (chitosan) and the coordinating reagent (polyphenolic compounds). Consequently, a higher release rate of polyphenolic compounds is observed in the simulated gastric fluid. In simulated small intestinal fluid, the release rate of polyphenolic compounds from CS-GTEMR was relatively low, reaching 3.375%. This finding is consistent with the research results obtained by Hameed et al. [38]. They prepared cross-linked chitosan microspheres encapsulating antiviral drugs using a copolymerization technique with polysaccharides extracted from pomegranate peel and chitosan. The swelling experiments demonstrated that the cross-linked chitosan microspheres could control the release of the encapsulated drug in a pH 7.4 solution, while the drug release rate was maximized at pH 1.3. In this study, within the pH range of 7–8, which simulates small intestinal fluid, the swelling ratio of CS-GTEMR reached its minimum value. In this weakly alkaline environment, where only a limited number of hydroxide ions are present, the amino groups of chitosan in CS-GTEMR remain unaffected by hydrogen ions. Consequently, the complexes formed between chitosan and polyphenolic compounds do not dissociate but instead maintain a stable existence. This protective mechanism ensures that the majority of polyphenolic compounds encapsulated within CS-GTEMR are effectively preserved and retained within the system. The cumulative release rate of polyphenolic compounds in simulated gastric fluid and simulated small intestinal fluid was 28.309%, indicating that nearly 72% of the polyphenolic compounds in CS-GTEMR can be further released and exert their active effects. In the large intestine environment, microorganisms can degrade or decompose the chitosan matrix of CS-GTEMR, allowing the retained polyphenolic compounds in CS-GTEMR to be completely released into the large intestine environment. There, they can exert their biological activities, such as antioxidant properties, to protect the health of the organism. Several studies have demonstrated that polyphenolic compounds, upon absorption, are widely distributed in tissues throughout the body, particularly exhibiting high concentrations in the esophagus, small intestine, and large intestine [39,40,41]. Consequently, a higher quantity of polyphenolic compounds in the large intestine corresponds to a greater content of these compounds in the colon, which is beneficial for the protective effects of polyphenols on colonic health.

### 3.4. Results and Analysis of Antioxidant Activity

Oxygen free radicals, including O_2_^−^, ·OH, ·OR, etc., are normal metabolites of human body metabolism. Under normal circumstances, the production and elimination of oxygen free radicals in the body are balanced. The presence of a small amount of oxygen free radicals in the human body can also promote cell proliferation and accelerate the bactericidal and anti-inflammatory effects of cells. However, once the production of oxygen free radicals in the body becomes excessive or the antioxidant system malfunctions, the metabolism of oxygen free radicals will be unbalanced, becoming an important factor causing aging and many diseases. For example, heart disease, hypertension, chronic pneumonia, etc., are all related to oxygen free radicals. Polyphenolic compounds exhibit antioxidant activity due to their ability to scavenge hydroxyl radicals and superoxide anions. Many plant extracts contain polyphenolic compounds, which can serve as natural food antioxidants, protecting the human body from the harmful effects of free radicals [42,43]. Alternatively, these compounds can be prepared into microspheres through coordination with some biopolymers and administered as a prophylactic agent via the digestive tract. This approach can increase the level of polyphenolic compounds in the digestive system, thereby playing a role in disease prevention.

The DPPH radical method is a rapid approach widely used to evaluate the ability of antioxidants to scavenge free radicals within a relatively short period of time. DPPH radical is a highly stable nitrogen-centered radical. If the tested compound can scavenge it, this indicates that the compound has the effect of reducing the effective concentration of hydroxyl radicals, alkyl radicals, or peroxy radicals, or interrupting the lipid peroxidation chain reaction. The DPPH radical ethanol solution is deep purple and exhibits a strong absorption peak near 517 nm. When a radical scavenger is added to the DPPH radical solution, the DPPH radical accepts electrons or hydrogen from the tested substance, transforming into a stable diamagnetic molecule. Consequently, the solution color changes from purple to yellow, and the absorbance value decreases. The magnitude of this decrease in absorbance is linearly related to the extent of radical scavenging. From this relationship, the IC_50_ value of the scavenger can be determined. Therefore, it can be used to detect the scavenging of DPPH free radicals, thereby evaluating the antioxidant properties of the sample. The smaller the IC_50_ value, the greater the scavenging rate and the stronger the antioxidant activity is. After adding CS-GTEMR to the DPPH solution, the optical density at 517 nm decreased rapidly, and the degree of color removal indicated the ability of CS-GTEMR to scavenge DPPH free radicals. Simultaneously, the scavenging abilities of crude tea polyphenols and ascorbic acid for DPPH free radicals were determined, and the results are shown in Table 3. CS-GTEMR has the ability to scavenge DPPH free radicals, and its effect is similar to that of crude tea polyphenols. Sathiyaseelan et al. encapsulated *Melaleuca alternifolia* oil within chitosan–sodium alginate microspheres and investigated the antioxidant, antibacterial, and wound healing properties of these microspheres [44]. The results indicated that the microspheres exhibited DPPH and ABTS radical scavenging abilities. Furthermore, the microspheres demonstrated negligible cytotoxicity and were found to promote the proliferation of NIH3T3 cells in an in vitro scratch assay. This suggests that the polyphenolic compounds encapsulated in CS-GTEMR are very stable and maintain good activity.

**Figure 7:**
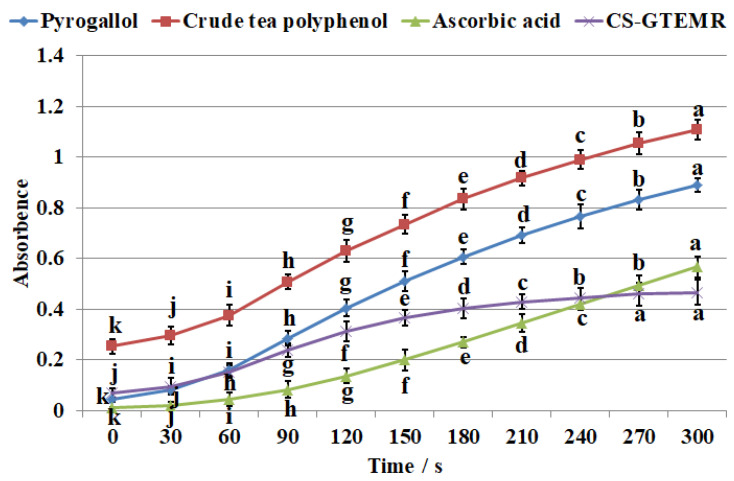
Scavenging superoxide anion radicals activities (different letters in each indicator in the same substance mean significant differences at 0.05 level (*p* < 0.05, *n* = 3)).

Pyrogallol undergoes auto-oxidation and decomposition in a weakly alkaline environment (pH 8.2) to produce O^−^_2_·. As the reaction progresses, O^−^_2_· accumulates in the system, resulting in a linear increase in the absorbance value of the reaction solution at a wavelength of 325 nm over time. By determining the rate of change in absorbance of the antioxidant reaction solution with time, the ability of the antioxidant to inhibit the accumulation of O^−^_2_· can be obtained. The kinetic curves for the auto-oxidation of pyrogallol, crude tea polyphenols, ascorbic acid, and CS-GTEMR in scavenging superoxide anion radicals are shown in Figure 7. The kinetic curves for the auto-oxidation of pyrogallol, crude tea polyphenols, and ascorbic acid in scavenging superoxide anion radicals all show a linear upward trend. However, the kinetic curve for CS-GTEMR in scavenging superoxide anion radicals flattens out after 240 s. No significant difference was observed in the absorbance values of the CS-GTEMR curve between 270 s and 300 s (*p* > 0.05). Furthermore, within the 0–300 s time range, significant differences were detected at all time points for the curves of pyrogallol, tea polyphenols, and ascorbic acid (*p* < 0.05). At the end of the reaction, the scavenging rate of CS-GTEMR for superoxide anion radicals was 52.893%, while the scavenging rate of ascorbic acid was 34.238%. This indicates that CS-GTEMR has good scavenging activity for superoxide anion radicals.

**Figure 8:**
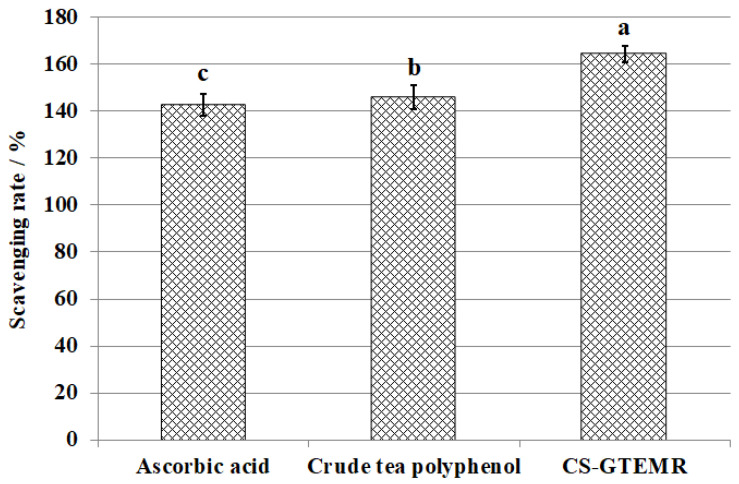
Scavenging hydroxyl radical activities (different letters in each substance mean significant differences at 0.05 level (*p* < 0.05, *n* = 3)).

Hydroxyl radicals are known as the strongest oxidant, capable of undergoing reactions such as dehydrogenation, addition, and electron transfer. These radicals can react with substances like amino acids, proteins, nucleic acids, and fats, causing oxidative damage to biological organisms. This damage can lead to aging and disease, posing significant harm to the body. Consequently, research on both the oxidative damage caused by hydroxyl radicals and their scavengers has garnered attention. The scavenging effects of ascorbic acid, crude tea polyphenols, and CS-GTEMR on hydroxyl radicals are illustrated in Figure 8. Compared to ascorbic acid and crude tea polyphenols, CS-GTEMR exhibits better scavenging activity against hydroxyl radicals (*p* < 0.05). Furthermore, crude tea polyphenols demonstrated slightly higher hydroxyl radical scavenging activity than ascorbic acid (*p* < 0.05). Zhu et al. prepared catechin-grafted chitosan and investigated its antioxidant activity [45]. Their findings indicated that at a concentration of 1 mg/mL, the catechin-grafted chitosan exhibited a reducing power of 0.51, a hydroxyl radical scavenging rate of 46.81%, and a DPPH radical scavenging rate of 67.08%. This study untangled that the antioxidant activity of catechin-grafted chitosan originates from the phenolic hydroxyl groups on the catechin molecules. This suggests that green tea extract encapsulated in chitosan microspheres can still retain its antioxidant activity.

**Figure 9:**
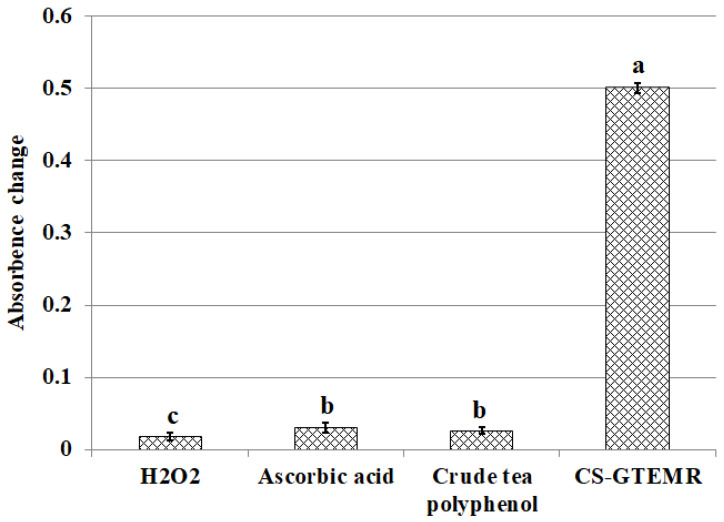
Scavenging hydrogen peroxide activities (different letters in each substance mean significant differences at 0.05 level (*p* < 0.05, *n* = 3)).

The determination of hydrogen peroxide scavenging activity is capable of assessing the ability of antioxidants to reduce peroxidizing agents. Although hydrogen peroxide itself does not exhibit high reactivity, its combination with superoxide anion can damage many cellular components. The results of hydrogen peroxide scavenging by ascorbic acid, crude tea polyphenols, and CS-GTEMR are presented in Figure 9. CS-GTEMR demonstrates the strongest effect on hydrogen peroxide, performing better than both ascorbic acid and crude tea polyphenols (*p* < 0.05). Furthermore, both ascorbic acid and crude tea polyphenol demonstrated superior absorbance variation trends compared to hydrogen peroxide (*p* < 0.05).

**Figure 10:**
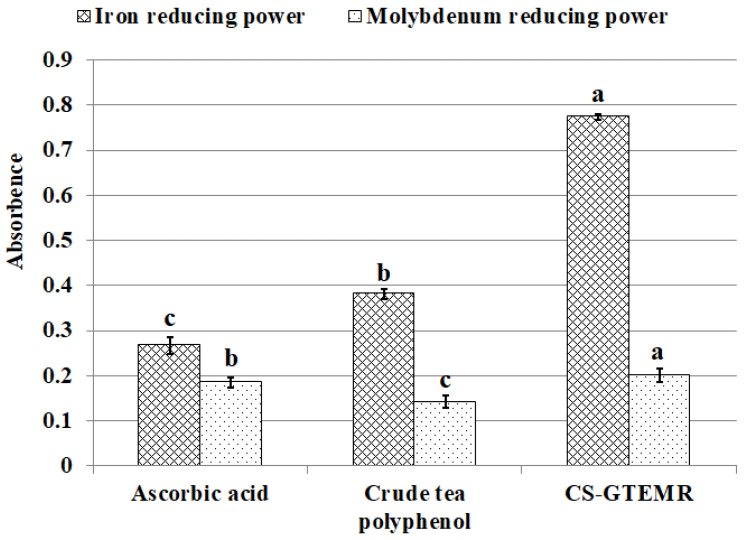
Iron and molybdenum reducing power activities (different letters in each substance in the same indicator mean significant differences at 0.05 level (*p* < 0.05, *n* = 3)).

Reducing power is a crucial indicator that represents the electron-providing capability of antioxidants. Antioxidants deactivate free radicals by donating electrons through their reducing action, thereby converting these radicals into stable molecules. Polyphenolic compounds, as antioxidants, possess the ability to donate electrons, and their antioxidant efficacy is closely linked to their reducing power. The stronger the reducing power, the greater the antioxidant activity. Figure 10 illustrates the results of iron-reducing power for ascorbic acid, crude tea polyphenols, and CS-GTEMR. Due to the presence of numerous aromatic ring hydroxyl groups, crude tea polyphenols exhibit a higher electron-donating capacity than ascorbic acid, resulting in a higher absorbance value (*p* < 0.05). As for CS-GTEMR, the absorbance value presented is obtained after diluting the reaction solution six times, indicating that its iron-reducing power significantly exceeds that of both ascorbic acid and crude tea polyphenols (*p* < 0.05). The phosphorus molybdenum blue method is based on the reduction of Mo(VI) to Mo(V) by antioxidant compounds, forming a green Mo(V) complex that has maximum absorption at 695 nm. Antioxidants block free radical chain reactions by donating hydrogen atoms. The results are shown in Figure 10. The absorbance value reflecting the molybdenum-reducing capacity of CS-GTEMR was significantly higher than that of crude tea polyphenols and ascorbic acid (*p* < 0.05). In conclusion, CS-GTEMR demonstrates superior reducing power activities.

## 4. Conclusions

This paper investigates the physical properties, structural characteristics, swelling behavior, polyphenol content, and antioxidant activity of CS-GTEMR prepared using the reversed-phase suspension cross-linking polymerization technique. The results indicated that CS-GTEMR exhibited a reddish-brown spherical shape with a porous, dense, and smooth surface. The average particle size is approximately 316.106 μm, and it contains a total polyphenol content of 50.485 μg/g. The presence of characteristic absorption peaks of phenolic hydroxyl groups in the FTIR spectrum suggests the existence of polyphenolic compounds within CS-GTEMR. The DSC results indicate that the thermal stability of CS-GTEMR is lower than that of chitosan powder and CS-MR. CS-GTEMR exhibits excellent swelling properties in both distilled water and solutions with pH ranging from 1.0 to 13.0. The cumulative release rate of polyphenolic compounds from CS-GTEMR in simulated gastric fluid and simulated intestinal fluid is only 28.309%, suggesting that the polyphenolic compounds within CS-GTEMR have the potential for further release and can exert antioxidant activities, such as scavenging free radicals and reducing power. The aforementioned results demonstrate that the successful encapsulation of green tea extract into the CS-GTEMR system was confirmed through quantitative determination of polyphenolic compounds and identification of the characteristic phenolic hydroxyl peak in FTIR spectroscopy. This confirms the successful preparation of chitosan microsphere materials with developmental potential for green tea extract protection. Furthermore, experimental data on swelling characteristics, controlled release performance in simulated gastrointestinal fluids, and antioxidant activity indicate that CS-GTEMR maintains stability across a wide pH range and achieves controlled release of polyphenolic compounds. Based on these findings, CS-GTEMR demonstrates potential for development as both a directly ingestible nutraceutical and pharmaceutical agent to safeguard human health. Furthermore, in food processing applications such as meat products, baked goods, and fruit/vegetable beverages, CS-GTEMR may serve as a promising natural antioxidant due to its controlled release of polyphenolic compounds. This property effectively inhibits lipid peroxidation, thereby contributing to extended food shelf life. In the future, various functional groups such as flavonoids, pigments, polysaccharides, and peptides could be grafted onto CS-GTEMR, enabling the development of chitosan microspheres with diverse functional activities. Subsequently, their applications may be extended to areas like encapsulation carriers for natural products, sustained-release agents for active substances, and clarification agents for fruit juices and beverages. The development and application of this range of CS-GTEMR products hold promise for playing a significant role in the food and beverage industry, the production of functional food ingredients, and the pharmaceutical industry. Therefore, it is crucial to further explore the activity and applications of the CS-GTEMR series of functional products in the future.

## Figures and Tables

**Table 1:** Physical properties of CS-GTEMR and CS-MR.

Physical Properties	CS-GTEMR	CS-MR [20]
Water absorption capacity/M (%)	64.296 ± 1.588	51.982 ± 1.944
Pile-up density/ρ_P_ (g/mL)	0.843 ± 0.087	0.862 ± 0.007
Skeletal density/ρ_T_ (g/cm^3^)	1.248 ± 0.480	1.212 ± 0.453
Pore degree/P	0.679 ± 0.075	0.554 ± 0.097
Free aldehyde group/(mmol/g)	0.267 ± 0.012	0.315 ± 0.009
Weak basic exchange capacity/(mmol/g)	1.409 ± 0.084	1.311 ± 0.084

**Table 2:** Main wave numbers of chitosan powder, CS-MR, and CS-GTEMR.

Peaks Attribution	Chitosan Powder (cm^−1^)	CS-MR (cm^−1^)	CS-GTEMR (cm^−1^)
ν(O-H) and ν(N-H)	3444.86	3420.21	3422.42
ν(-CH_3_)	2917.82	2925.88	2925.15
ν(-CH_2_)	2875.65	2875.98	2862.14
ν(-CHO)	-	1715.07	1712.62
amide I (ν(C=O) and ν(C-N))	1649.84	1650.65	1650.48
δ(-NH_2_)	1597.87	-	-
ν(C=N)	-	1573.36	1558.52
amide II (δ(N-H) and ν(C-N))	1540.99	-	1541.28
amide III (ν(C-N) and δ(N-H))	1259.77	1227.50	1227.18
ν(C-O-C)	1154.80	1102.21	1103.10
C_6_-OH (ν(C-O))	1087.55	weaker	weaker
C_3_-OH (ν(C-O))	1029.13	1031.00	1024.82
characteristic absorption peak of β-D-glucopyranoside	897.47	weaker	916.50
phenolic hydroxyl group (ν(H-O))	-	-	1230.00

**Table 3:** Results of scavenging DPPH free radicals.

Peaks Attribution	CS-GTEMR	Crude Tea Polyphenol	Ascorbic Acid
IC_50_	0.16 g/mL	61.49 μg/mL	70.66 μg/mL
scavenging rate/%	59.42 ± 3.99	64.78 ± 1.46	40.35 ± 4.44

## Data Availability

The original contributions presented in this study are included in the article. Further inquiries can be directed to the corresponding author.
